# Centrosomal protein 72 deficiency exacerbates liver fibrosis induced by *Schistosoma japonicum* infection

**DOI:** 10.1186/s13071-026-07372-7

**Published:** 2026-05-05

**Authors:** Qian Fang, Guangbo Mei, Xuejun Zhao, Weijia Xu, Shanshan Li, Yinan Wang, Zhe Mao, Jiaxi Zhang, Kejun Liu, Jiayi Feng, Wenjuan Yang, Xuebing Qiu, Na Kuang, Hong Jiang, Xiaoqing Li, Rui Zhou, Huifen Dong, Zhenping Ming

**Affiliations:** 1 Hubei Province Key Laboratory of Allergy and Immunology, Taikang Medical School, School of Basic Medical Sciences, Wuhan University, Wuhan, 430071 Hubei China; 2 Department of Medical Parasitology, School of Basic Medical Sciences, Wuhan University, Wuhan, 430071 Hubei China; 3 Kingstar Global, Wuhan, 430070 China; 4 Snail Ecological Station of Gongan County, Jingzhou, 434300 China; 5 Center for Stem Cell Research and Application, Union Hospital, Tongji Medical School, Huazhong University of Science and Technology, Wuhan, Hubei 430022 People’s Republic of China

**Keywords:** *Schistosoma japonicum*, Liver fibrosis, Centrosomal protein 72, CCl_4_

## Abstract

**Background:**

Hepatic fibrosis induced by *Schistosoma japonicum* (*S. japonicum*) infection is a major global public health concern. Centrosomal protein 72 (CEP72), a key regulator involved in maintaining cellular architecture and integrity, is significantly upregulated during the progression of hepatic fibrosis; however, its specific biological function in this pathological process remains largely elusive. This study was designed to elucidate the novel biological role of CEP72 in liver fibrosis, with a particular focus on the pathogenesis of *S. japonicum*-induced hepatic fibrosis.

**Methods:**

Publicly available transcriptomic datasets of human hepatic fibrosis were analyzed, and the key findings were validated in two murine models of liver fibrosis (*S. japonicum* infection and carbon tetrachloride (CCl_4_) injection). To investigate the functional role of CEP72 in hepatic fibrogenesis, *Cep72* knockout (*Cep72*^−/−^) mice were employed. Histological staining was performed to evaluate liver pathological changes, fibrotic area, and granuloma size. Transcriptomic profiling, quantitative real-time reverse transcription polymerase chain reaction (qRT-PCR), immunohistochemistry (IHC), and western blot analyses were performed to assess the fibrogenic and inflammatory responses in liver tissues.

**Results:**

CEP72 expression was significantly elevated in both human fibrotic liver samples and murine models of hepatic fibrosis. Notably, CEP72 deficiency markedly exacerbated liver fibrosis, as evidenced by significantly increased granuloma size and enhanced collagen deposition in both *S. japonicum*-infected and CCl_4_-treated mice. Transcriptomic analysis revealed a global upregulation of pro-fibrotic and pro-inflammatory genes in the livers of *Cep72*^−/−^ mice compared with wild-type controls. These findings were further confirmed by qRT-PCR, IHC, and western blot analyses, which showed increased expression of fibrogenic markers, including α-smooth muscle actin and Collagen I. Mechanistically, loss of CEP72 promoted hepatic fibrogenesis by enhancing the expression of the transcription factor early growth response 1 (EGR1), which in turn upregulated tumor necrosis factor-α (TNF-α) transcription.

**Conclusions:**

Collectively, our findings demonstrate that CEP72 functions as a key negative regulator of inflammation-driven hepatic fibrosis. CEP72 deficiency accelerates the progression of liver fibrosis through the EGR1–TNF-α signaling pathway. This study identifies a previously unrecognized protective role of CEP72 in hepatic fibrosis and highlights its potential as a novel therapeutic target for the treatment of *S. japonicum*-induced and other types of inflammation-associated liver fibrosis.

**Graphical Abstract:**

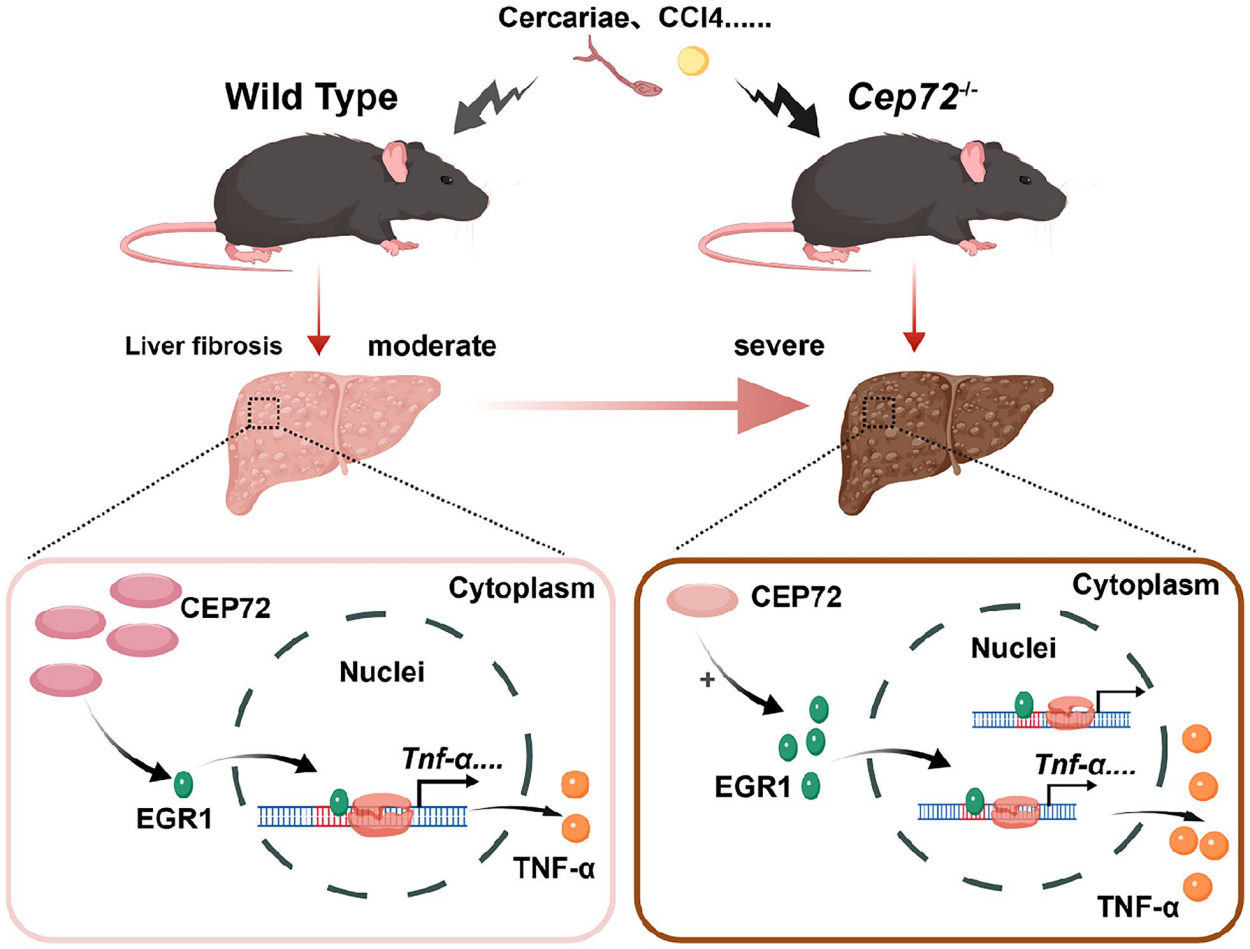

**Supplementary Information:**

The online version contains supplementary material available at 10.1186/s13071-026-07372-7.

## Background

Schistosomiasis is prevalent in 78 countries, primarily in tropical and subtropical regions, and is mainly caused by the species *S. japonicum*, *S. mansoni*, and *S. haematobium* [1]. According to the latest official statistics from the World Health Organization (WHO), schistosomiasis affects nearly 250 million people worldwide, with more than 700 million living in endemic areas [2]. Among these species, *S. japonicum* produces the most eggs and poses the most serious risk of liver fibrosis, which is the main pathological change associated with schistosomiasis [3]. Liver fibrosis can ultimately lead to portal hypertension, the leading cause of morbidity and mortality related to *S. japonicum* infection [4]. In addition to *S. japonicum* infection, other chronic liver diseases such as viral hepatitis, nonalcoholic steatohepatitis (NASH), alcoholic liver disease (ALD), and autoimmune diseases (AID) can also cause liver fibrosis [5–7]. The pathological process of these liver fibrosis types is characterized by fibrogenesis, which is driven by signals from stressed or injured hepatocytes and activated macrophages. This leads to activation of resident hepatic stellate cells (HSCs) into myofibroblasts, causing them to produce extracellular matrix (ECM) faster than it can be degraded [8]. Without effective intervention and treatment, liver fibrosis can progress into cirrhosis or even hepatocarcinoma. However, the molecular mechanisms regulating *S. japonicum*-induced liver fibrosis are not yet fully understood.

Centrosomal proteins are integral components of the centrosome and centriolar satellites and are essential for centriole biogenesis, microtubule organization, and cell-cycle progression [9]. To date, more than 30 centrosome proteins have been reported [10]. Several members of the CEP family have been tested, playing important role in the pathogenesis of liver diseases. For instance, CEP55 is associated with the growth, proliferation, and metastasis of hepatocarcinoma [11]. CEP350, another member of the CEP protein family, which interacts with peroxisome proliferator-activated receptor alpha (PPARα), has been reported to be targeted by miR-C12, thereby inhibiting RNA replication in viral hepatitis C [12]. Beyond cell division, the mother centriole can function as a basal body to nucleate the primary cilium, a sensory organelle that coordinates multiple signaling pathways implicated in tissue homeostasis and repair. In the liver, primary cilia are present on several cell types, and profibrotic cues can disrupt ciliary homeostasis in hepatic stellate cells (HSCs), thereby promoting HSC activation and liver fibrosis [13]. In addition, cilia-dependent signaling such as Hedgehog signaling has been implicated in chronic liver injury and fibrogenic remodeling [14]. Centrosomal protein 72 (CEP72) is a centriolar satellite protein that localizes to the centrosome. It is indispensable for core centrosomal functions, including cell division, microtubule organization, and faithful chromosome segregation [15]. Importantly, CEP72 interacts with pericentriolar material 1 (PCM1) and centrosomal protein 290 (CEP290) and is required for efficient trafficking/recruitment of Bardet–Biedl syndrome complex (BBSome)-associated components during ciliogenesis, supporting a role in primary cilium regulation [9]. Intriguingly, marked high expression of CEP72 in liver fibrosis caused by schistosomiasis, hepatitis B, and CCl_4_ were found recently in our laboratory, with poor understanding of the role of CEP72 in liver fibrosis. In this study, we further explored the functions of CEP72 in the pathogenesis of liver fibrosis caused by *S. japonicum* infection and its possible relational mechanism. The research will provide novel insights into the underlying mechanism of CEP72 in liver fibrosis.

## Methods

### Establishment of the hepatic fibrosis model using *S. japonicum* or CCl_4_

Eight-week-old female C57BL/6 J mice were purchased from the Hubei Provincial Center for Disease Control and Prevention (Wuhan, China). *Cep72* knockout (*Cep72*^−/−^) mice were gifts from Dr.M.C Luo (Wuhan University). With specific primers *Cep72*-F (5′-TACAGTATCTGGTCTCCTTGG-3′) and *Cep72*-R (5′-CATGGAACAGCAGCATAATC-3′), the mice with 218-bp or 251-bp PCR products were confirmed as *Cep72*^−/−^ transgene mice or wild-type (WT) mice respectively. *Oncomelania hupensis* snails were provided by the Snail Ecological Station of Gong’an County (Jingzhou, China). Liver fibrosis models were established in C57BL/6 J mice either by abdominal infection with 17 ± 1 *Schistosoma japonicum* cercariae, with tissues collected 8 weeks post infection, or by intraperitoneal (i.p.) injection of CCl_4_ (Sigma-Aldrich; 10 mL/kg body weight, diluted 1:4 in olive oil and filtered) twice weekly for 8 weeks, followed by tissue collection [16, 17]. All animal experiments were conducted in accordance with the guidelines of the Animal Protection and Use Committee of Wuhan University and the rules and regulations of Wuhan University (approval no. WP20260029).

### RNA extraction and real-time PCR

Total RNA was extracted from liver tissues using TRIzol reagent according to manufacturer’s instruction. Reverse transcription was performed using a complementary DNA (cDNA) Reverse Transcription Reagent Kit (Vazyme, China). The expression level of *Cep72*, *Acta2*, *Col1a1*, *Col3a1*, *Tgfβ1*, *Des*, *Timp1*, *Egr1*, *Tnf-α*, *Cxcl10*, *Actin*, and *Gapdh* was detected using the SYBR Green Master Mix Kit (Vazyme, China), and fold changes were calculated using the 2^−ΔΔCt^ method. *Gapdh* and *Actin* was used as endogenous control genes. The gene primers were designed by Primer Premier 5. The primers used in this study are listed in Supplementary Table 1.

### Western blotting

Total protein was extracted from liver tissues with radioimmunoprecipitation assay (RIPA) lysis buffer (Servicebio, China) and quantified by the Bradford method (Biosharp, China). Cell lysates were then loaded in sodium dodecyl sulfate (SDS)-polyacrylamide gel electrophoresis (PAGE) gel to separate proteins and transferred to 0.45-μm polyvinylidene fluoride (PVDF) membranes (Millipore, USA). The membranes were blocked with 5% non-fat milk in Tris-buffered saline with Tween 20 (TBST) for 1 h, and then incubated with the corresponding primary antibodies overnight at 4 °C. After three washes with TBST, the membranes were incubated with horseradish peroxidase (HRP)-conjugated secondary antibodies for 1 h at room temperature. Following three additional TBST washes, the membranes were incubated with enhanced chemiluminescence (ECL) Western blot Substrate (Vazyme, China) and exposed to X-Ray Super RX Films (Fujifilm, Japan) for detection. Antibodies to ACTA2 (Proteintech, 14395–1-AP), COL1A1 (Proteintech, 67288–1-AP), GAPDH (Proteintech, 60004), and EGR1 (HUABIO, ET7111-41) were used for blotting.

### Hydroxyproline content analysis

The levels of hydroxyproline content in liver tissue was measured by using hydroxyproline kit (Nanjing Jiancheng Bioengineering Institute, China).

### AST and ALT analysis

The serum levels of alanine aminotransferase (ALT) and aspartate aminotransferase (AST) were measured by using ALT Assay Kit and AST Assay Kit (Nanjing Jiancheng Bioengineering Institute, China).

### Histological and immunohistochemistry analysis

Liver tissues were fixed in 4% formalin at room temperature, continuously dehydrated in ethanol, and embedded in paraffin. Paraffin sections were stained with Meyer hematoxylin and stained with eosin (H&E). Representative areas of each stained tissue section were imaged at 40× magnification. The experimental method of Masson staining was based on a Masson staining kit. For immunohistochemistry, tissue sections were incubated with the antibodies against EGR1 (Starter, S0B6029), ACTA2 (Proteintech, 14395–1-AP), COL1A1 (Proteintech, 67288–1-AP), CEP72 (Proteintech, 19928–1-AP), and TGF-β1 (HUABIO, HA721143), for 1 h at room temperature. These slides were then subjected to horseradish peroxidase-linked secondary antibodies for 1 h at room temperature. The sections were visualized by using the 3,3′-diaminobenzidine (DAB) substrate kit (Servicebio, China) and representative areas of each stained tissue section were imaged at 100× magnification. ImageJ software was used to quantify the staining results.

### Plasmid DNA construction

The upstream regions of the *CXCL10* and *TNF-α* genes were amplified by PCR and then cloned into the luciferase vector, pGL3-basic, to generate the reporter plasmids. The coding regions of *EGR1* were amplified by PCR and cloned into the overexpression vector pcDNA3.1. The primers are listed in Supplementary Table 1.

### Luciferase reporter assay

In this assay, 293T cells were seeded in a 24-well plate at a density of 1 × 10^5^ cells per well, 24 h prior to transfection. The pGL3-basic reporter vectors containing the *TNF-α* and *CXCL10* promoter fragment were then transfected using 0.5 μL of Neofect™ DNA transfection reagent (Neofect, China), following the manufacturer’s instructions. After 24 h of transfection, the cells were harvested and lysed using reporter lysis buffer (Vazyme, China). Luciferase activities were measured using a Luciferase Assay System (Promega, USA), and the firefly luciferase activities were normalized to the *Renilla* luciferase activities.

### RNA-seq and analysis

Total RNA was extracted from liver tissues using TRIzol reagent according to manufacturer’s instruction, and RNA quality was checked using an Agilent Bioanalyzer 2100. The mRNA was purified using KAPA mRNA Capture Kits (Roche), and cDNA libraries were prepared using KAPA RNA HyperPrep Kits (Roche) at Kindstar Global (Wuhan, China). Equal amounts of cDNA library from each sample were pooled for sequencing on an Illumina HiSeq X platform (150-bp paired-end sequencing). Reads were mapped to the GRCm38 genome assemblies using Hisat2 v2.1.0 with the default settings. The aligned reads were converted to bigwig coverage files using reads per million. Deseq2 were used for differentially expressed gene analysis.

### Statistical analysis

Statistical analyses were performed using WPS Office and GraphPad Prism 8.0. Data are expressed as mean ± standard deviation (SD)/standard error of the mean (SEM). Comparisons between two groups were carried out using an unpaired two-tailed Student’s *t*-test, and comparisons among multiple groups were performed using one-way analysis of variance (ANOVA) with appropriate post hoc tests, as indicated in the figure legends. The number of biological replicates for each experiment is specified in the corresponding figure legends. A value of *P* < 0.05 was considered statistically significant.

## Results

### CEP72 expression is upregulated in murine model of liver fibrosis and in human *S. japonicum*-infected liver

**Fig. 1:**
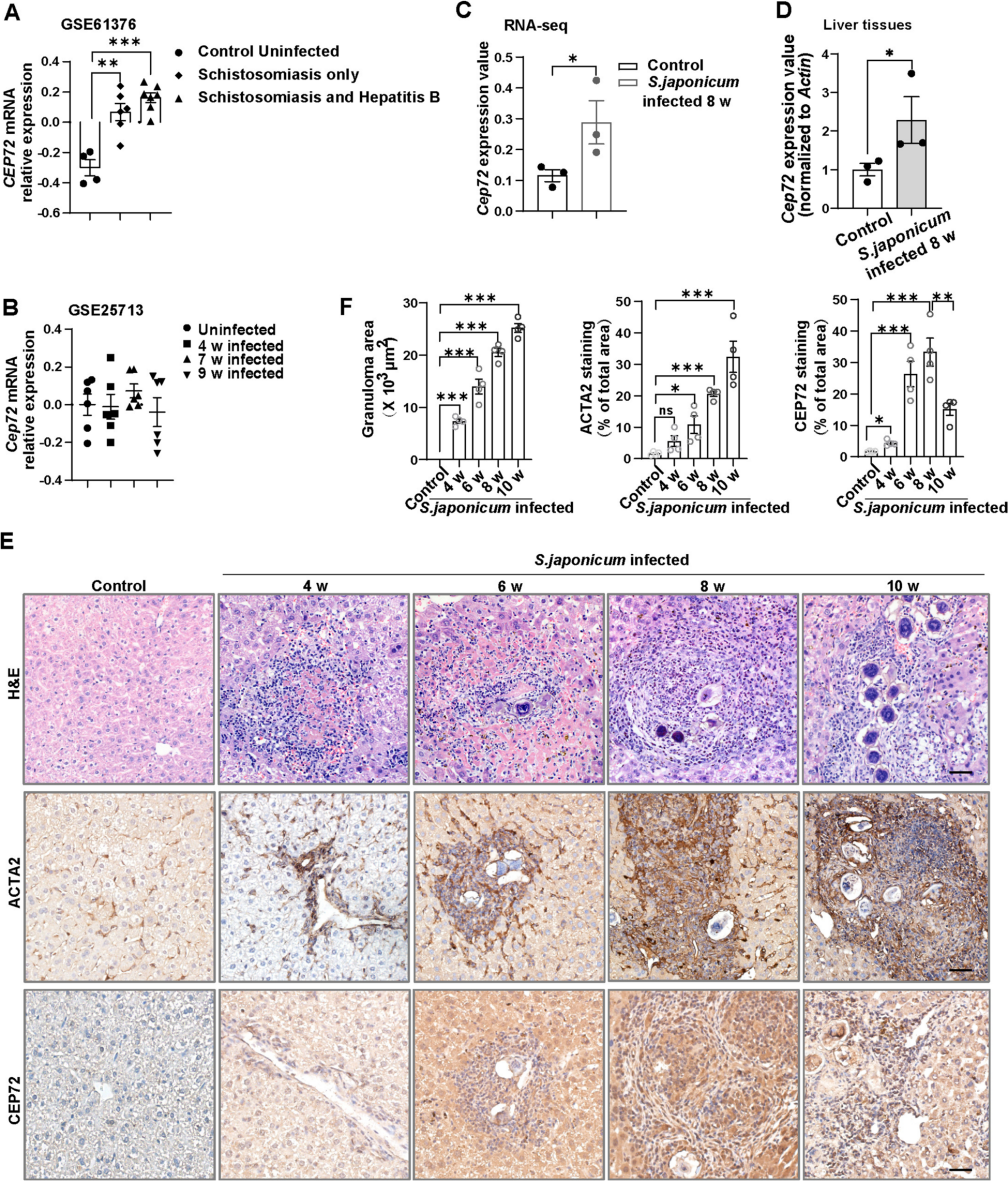
CEP72 expression is upregulated in murine model of liver fibrosis and in human *S. japonicum*-infected liver. **A** Expression of *CEP72* in clinical samples from GSE61376, including schistosomiasis (*n* = 6), schistosomiasis and hepatitis B co-infected (*n* = 7), and control (*n* = 4) groups. **B** *Cep72* expression was evaluated in a mouse model of *S. japonicum* infection at 7 and 9 weeks post-infection (*n* = 6 per group), alongside uninfected controls (*n* = 6), using data from GSE25713. **C** *Cep72* transcript levels were assessed using an in-house RNA-seq data obtained from fibrotic mouse liver tissues following 8-week *S. japonicum* infection. **D** *Cep72* mRNA expression level was assessed in liver tissues from uninfected mice and mice infected with *S. japonicum* for 8 weeks by qRT-PCR. **E** H&E staining, ACTA2 staining, and CEP72 staining (all scale bars, 50 μm) of liver sections from the indicated groups (uninfected, 4 w/infected, 6 w/infected, 8 w/infected, and 10 w/infected). **F** Graphs show the quantified positive areas for each stain, determined using ImageJ software from multiple randomly selected fields across distinct tissue sections. Data are presented as mean ± SEM. Statistical analysis was performed using one-way ANOVA followed by Tukey’s post hoc test for multiple-group comparisons (**A**, **B**, **F**) and unpaired two-tailed Student’s t-test for two-group comparisons (**C**, **D**). *P < 0.05; **P < 0.01; ***P < 0.001; ****P < 0.0001; ns, not significant.

To investigate the alterations in CEP72 expression in liver fibrosis, we initially analyzed previously published genome-wide expression profiling data from patients with schistosomiasis-infected liver fibrosis and control subjects. The mRNA expression of *CEP72* showed upregulation in schistosomiasis patients compared with those of controls (GSE61376) (Fig. 1A). Publicly available mouse transcriptome datasets also indicate a similar upregulation of *Cep72* mRNA expression in liver *fibrosis* induced by *S. japonicum* (GSE25713) (Fig. 1B). An in-house RNA-seq dataset, generated by our research group from fibrotic mouse liver tissues at advanced fibrosis stages induced by 8 weeks of *S. japonicum* infection, and quantitative real-time reverse transcription polymerase chian reaction (qRT-PCR) results further confirmed the upregulation of *Cep72* mRNA expression in fibrotic liver tissues (Fig. 1C, D). Notably, H&E staining and immunohistochemistry staining analysis collectively demonstrated that CEP72 expression commenced increasing in *S. japonicum*-infected mouse livers at 4 weeks post-treatment and continued to rise up to 8 weeks, exhibiting a positive correlation with the progression of hepatic fibrosis (Fig. 1E, F).

To assess whether this upregulation of *Cep72* is specific to schistosomiasis-associated fibrosis or represents a more general response to chronic liver injury, we next examined its expression in a classical CCl_4_-induced liver fibrosis model. Consistent with the findings in *S. japonicum*-induced liver fibrosis, publicly available mouse transcriptome datasets showed an upregulation of *Cep72* mRNA expression in CCl_4_-induced liver fibrosis at 6 and 8 weeks (GSE222576) (Supplementary Fig. S1A). Likewise, our in-house RNA-seq dataset confirmed that *Cep72* mRNA expression was increased in fibrotic livers from mice treated with CCl_4_ for 8 weeks (Supplementary Fig. S1B). Further experiments conducted on mice following CCl_4_ induction for 8 weeks revealed that, compared with control mice, the expression levels of CEP72 mRNA and protein were both significantly upregulated, as determined by qRT-PCR and IHC analysis (Supplementary Fig. S1C, D). Taken together, these findings indicate that CEP72 is consistently upregulated during the development of liver fibrosis.

### CEP72 deficiency aggravates *S. japonicum*- or CCl_4_- induced liver fibrosis

**Fig. 2:**
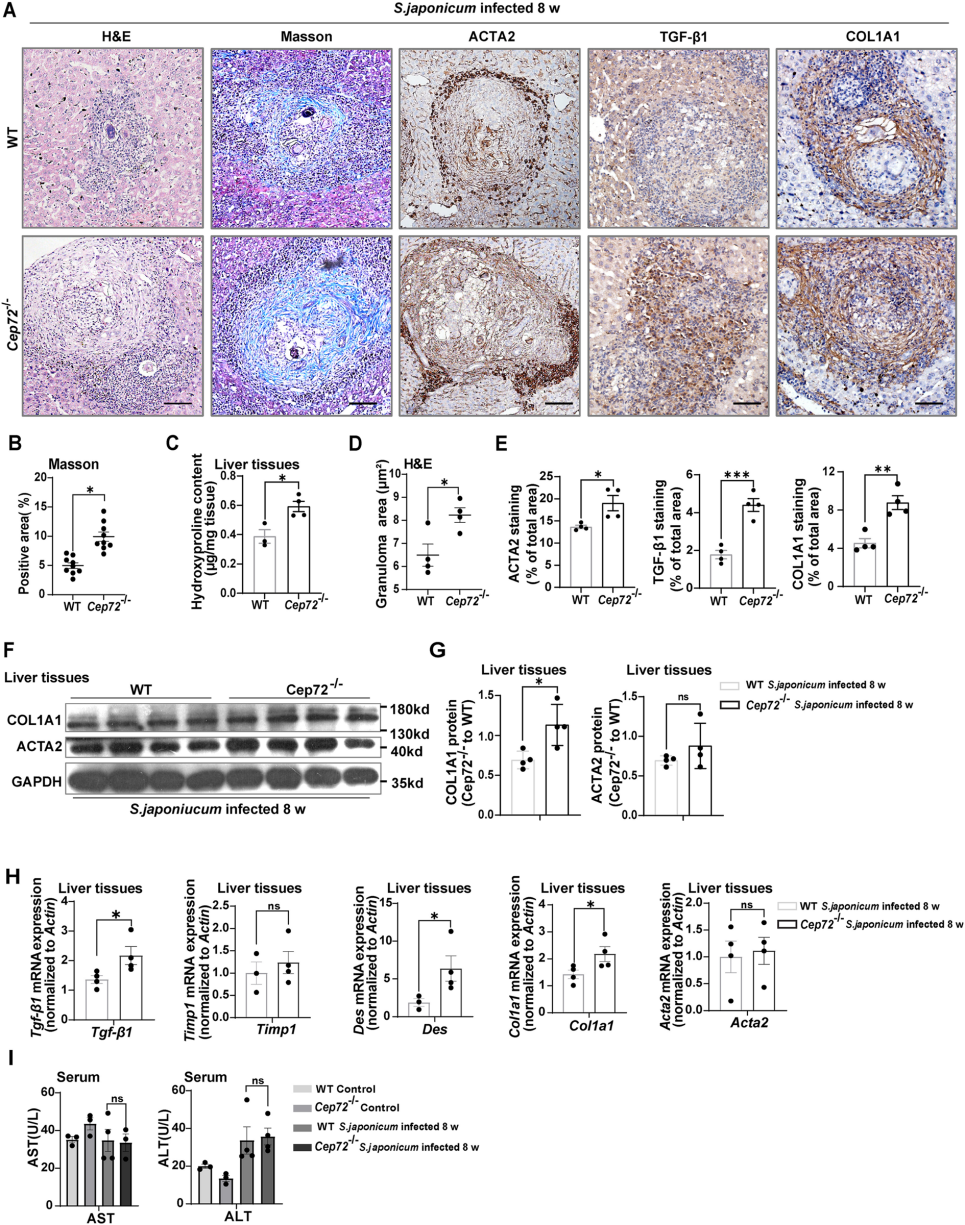
CEP72 deficiency aggravates *S. japonicum*-induced liver fibrosis. **A** Representative H&E, Masson, and immunohistochemical staining for ACTA2, TGFβ-1, and COL1A1 in liver sections from WT and *Cep72*^−/−^ 8 weeks after *S. japonicum* infection (scale bars, 50 μm). **B** Quantification of Masson-positive areas in liver sections, analyzed using ImageJ from multiple randomly selected fields. **C** Hepatic hydroxyproline content. **D** Granuloma area measured from H&E-stained liver sections. **E** Quantification of ACTA2, TGF-β1, and COL1A1 immunohistochemical staining in liver sections. **F**, **G** Western blot analysis of COL1A1 and ACTA2 in liver tissues from WT and *Cep72*^−/−^ mice 8 weeks after *S. japonicum* infection (**F**), with densitometric quantification of protein levels (**G**). **H** Hepatic mRNA expression of *Col1a1*, *Tgf-β1*, *Acta2*, *Timp1*, and *Des* determined by qRT-PCR. **I** Serum ALT and AST levels in WT and *Cep72*^−/−^ mice with or without *S. japonicum*-infection. Data are presented as mean ± SD. Sample sizes were as follows: WT control (*n* = 3), Cep72^−/−^ control (*n* = 3), WT *S. japonicum*-infected (*n* = 4), and Cep72^−/−^ *S. japonicum*-infected (*n* = 4). The results shown are representative of three independent experiments. Statistical analysis was performed using an unpaired two-tailed Student’s *t*-test. *P < 0.05; **P < 0.01; ***P < 0.001; ****P < 0.0001; ns, not significant

To investigate the functional role of CEP72 in liver fibrosis, we used *Cep72* knockout (*Cep72*^−/−^) mice, which were obtained from a previously established line and verified by PCR-based. The qRT-PCR and IHC staining results demonstrated that CEP72 expression in *Cep72*^−/−^ mice was significantly decreased compared with WT mice (Supplementary Fig. S1D, F). Despite this reduction, *Cep72*^−/−^ mice showed no evidence of basal liver damage or inflammatory cell infiltration, as indicated by H&E staining compared with the control group (Supplementary Fig. S2E). We then used *Cep72*^−/−^ mice to establish murine models of liver fibrosis induced by *S. japonicum* infection or CCl_4_ induction. Following *S. japonicum* infection, H&E and Masson’s staining revealed that loss of *Cep72* in *S. japonicum*-induced mice significantly aggravated hepatic fibrosis compared to controls (Fig. 2A, B). This was further confirmed by a significant increase in the level of hydroxyproline and the area of egg granuloma (Fig. 2C, D). Furthermore, the IHC staining demonstrated increased expression of fibrosis marker, including alpha-smooth muscle actin (α-SMA or ACTA2), collagen type I alpha 1 (COL1A1), and transforming growth factor-β1 (TGF-β1) in the livers of *Cep72*^−/−^ mice following *S. japonicum* infection, compared with controls (Fig. 2A, E). qRT-PCR and Western blot further confirmed an upregulation of fibrosis-related factors at both the mRNA and protein levels in the livers of *Cep72*^−/−^ mice compared with controls (Fig. 2F–H). In contrast, neither the serum AST or ALT showed significant difference between WT and *Cep72*^−/−^ mice after *S. japonicum* infection (Fig. 2I).

**Fig. 3:**
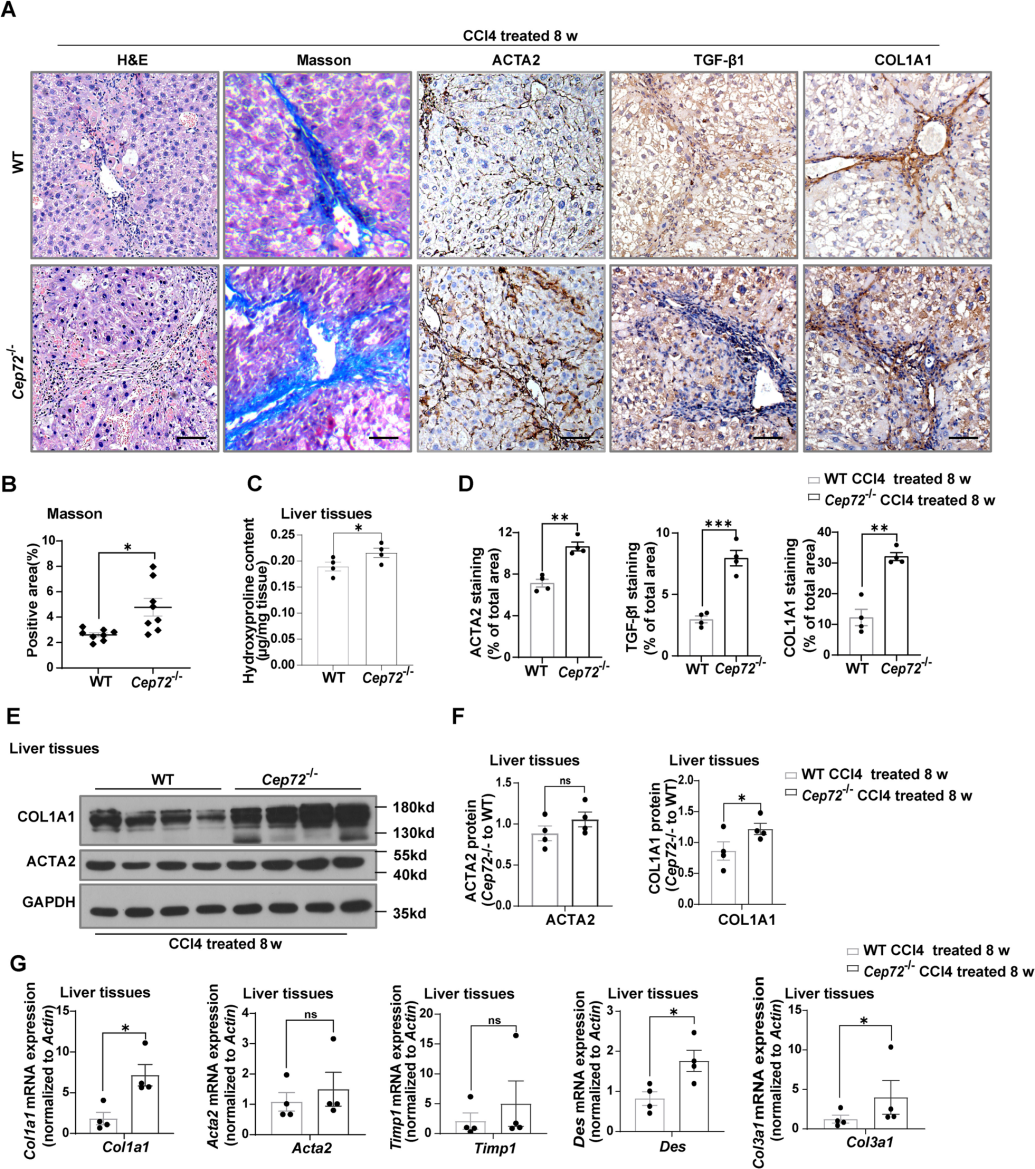
CEP72 deficiency aggravates CCl_4_-induced liver fibrosis. **A** Representative H&E, Masson, and immunohistochemical staining for ACTA2, TGF-β1, and COL1A1 in liver sections from WT and *Cep72*^−/−^ mice after 8 weeks of CCl_4_ treatment (scale bars, 50 μm). **B** Quantification of Masson-positive areas in liver sections, analyzed using ImageJ from multiple randomly selected fields. **C** Hepatic hydroxyproline content. **D** Quantification of ACTA2, TGFβ1, and COL1A1 positive areas from immunohistochemical staining, analyzed using ImageJ from multiple randomly selected fields. **E**, **F** Western blot analysis of COL1A1 and ACTA2 in liver tissues from WT and *Cep72*^−/−^ mice after 8 weeks of CCl_4_ treatment (**E**), with densitometric quantification of protein levels (**F**). **G** Hepatic mRNA expression of *Col1a1*, *Timp1*, *Acta2*, *Col3a1*, and *Des* determined by qRT-PCR. Data are presented as the mean ± SD (*n* = 4 mice per group) and are representative of three independent experiments. Statistical analyses were performed using an unpaired two-tailed Student’s *t*-test. *P < 0.05; **P < 0.01; ***P < 0.001; ****P < 0.0001; ns not significant

Additionally, a widely used CCl_4_-induced liver fibrosis model was employed to further investigate the role of CEP72 in liver fibrosis. Consistent with the findings in the *S. japonicum* infection model, *Cep72*^−/−^ mice subjected to CCl_4_ administration exhibited more severe fibrosis than control mice, histopathology (H&E and Masson’s staining), immunohistochemistry, hepatic hydroxyproline content, western blotting, and quantitative analysis of fibrosis marker genes (Fig. 3A–G). Together, these data indicate that loss of *Cep72* aggravates liver fibrosis in both *S. japonicum*- and CCl_4_-induced mouse models.

### Pro-fibrotic and pro-inflammatory genes were upregulated in *Cep72*^−/−^ mice according to RNA-seq

**Fig. 4:**
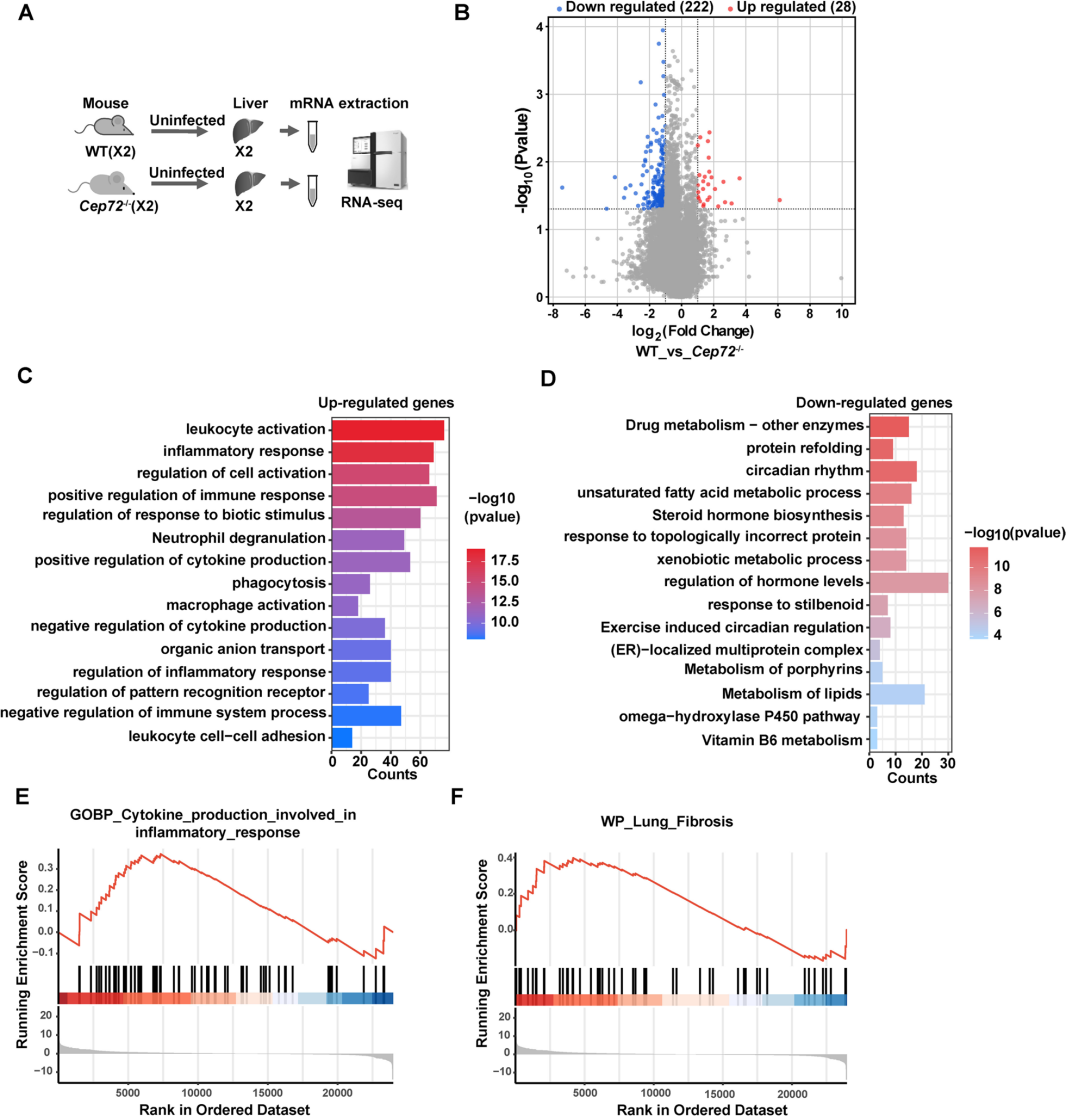
Pro-fibrotic and pro-inflammatory genes were upregulated in *Cep72*^−/−^ mice according to RNA-seq. **A** Schematic diagram of the experimental design. Liver samples from WT and *Cep72*^−/−^ mice (*n* = 2 per genotype) were collected for RNA-seq. **B** Volcano plot showing differentially expressed genes (DEGs) between WT and *Cep72*^−/−^ livers. **C**, **D** Pathway enrichment analysis of upregulated (C) and downregulated (**D**) DEGs identified by RNA-seq. **E**, **F** Gene set enrichment analysis (GSEA) of cytokines production involved in inflammatory response (**E**) and lung fibrosis (**F**) in *Cep72*^−/−^ versus WT livers

To gain further insight into the function of CEP72 in the liver, we performed high-throughput RNA sequencing (RNA-seq) on liver tissues from WT and *Cep72*^−/−^ mice (Fig. 4A). Differential gene analysis between WT and *Cep72*^−/−^ groups exhibited 222 downregulated genes and 28 upregulated genes, respectively (adjusted *p* < 0.05, log_2_ fold change ≥ 1; Fig. 4B). Gene Ontology (GO) analysis revealed that upregulated genes were highly involved in inflammatory response, leukocyte activation, and immune response (Fig. 4C). By contrast, the downregulated genes were highly enriched in drug metabolism, protein refolding, and circadian rhythm (Fig. 4D). Gene set enrichment analysis (GSEA) confirmed the inflammatory response activation, especially cytokines production involved in inflammatory response, in *Cep72*^−/−^ mice (Fig. 4E). Moreover, we also focused on the fibrosis-related response. Similarly, GSEA analysis showed that lung fibrosis-related genes were upregulated in *Cep72*^−/−^ mice (Fig. 4F). In conclusion, these findings confirm that CEP72 deletion promotes expression of fibrotic and inflammatory genes.

### CEP72 deficiency activates the expression of several fibrotic genes in *S. japonicum-* and CCl_4_-induced liver fibrosis

**Fig. 5:**
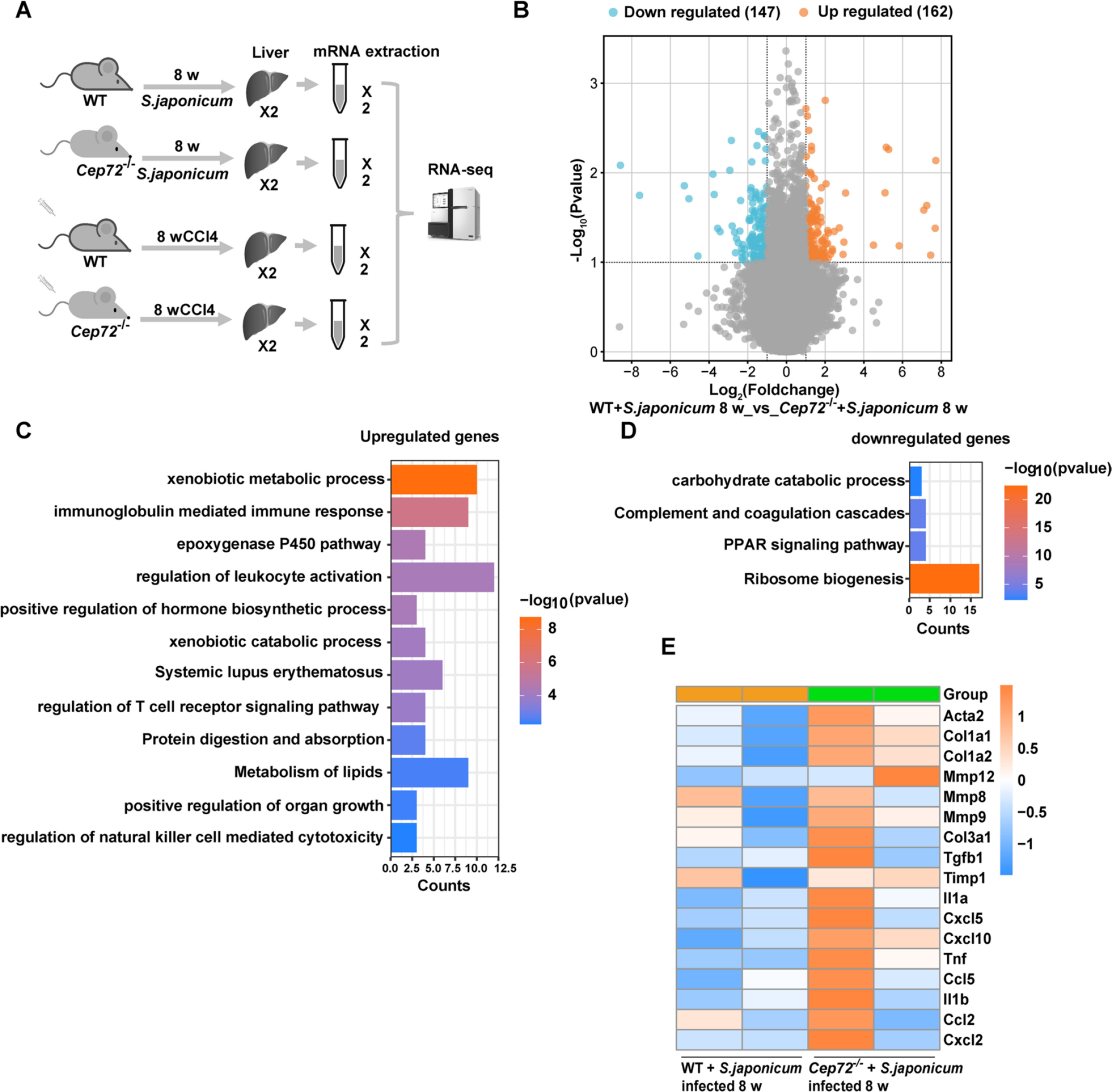
Pro-fibrotic and pro-inflammatory genes were upregulated in *Cep72*^−/−^ mice after *S. japonicum* infection according to RNA-seq. **A** Schematic diagram of the experimental design. Liver samples from WT and *Cep72*^−/−^ (n = 2 per genotype) subjected to *S. japonicum* infection or CCl_4_ treatment were collected for RNA sequencing; the analyses shown here correspond to the *S. japonicum* cohort. **B** Volcano plot showing differentially expressed genes between WT and *Cep72*^−/−^ livers after *S. japonicum* infection. **C**, **D** Pathway enrichment analysis of upregulated (**C**) and downregulated (**D**) genes identified by RNA-seq in WT and *Cep72*^−/−^ mice following *S.japonicum* infection. **E** Heatmap displaying the expression patterns of fibrosis-related genes and inflammatory mediators in WT and *Cep72*^−/−^ livers after *S. japonicum* infection

To further investigate the mechanism of CEP72 in liver fibrosis, RNA-seq was performed to analyze the livers of WT and *Cep72*^−/−^ mice after *S. japonicum* infection or CCl_4_ treatment (Fig. 5A). Compared with WT control mice, 147 downregulated genes and 162 upregulated genes after *S. japonicum* infection were identified in *Cep72*^−/−^ mice (adjusted *p* < 0.1, log_2_ fold change ≥ 1; Fig. 5B). GO analysis revealed that the upregulated genes tend to be highly enriched in the xenbiotic metabolic process and immunoglobulin mediated immune response (Fig. 5C), whereas downregulated genes were mainly involved in the ribosome biogenesis and PPAR signaling pathway (Fig. 5D). Transcriptomic analyses revealed that fibrosis-related genes, including *Acta2*, *Col1a1*, *Col1a2*, and *Mmp9*, were remarkably increased in *Cep72*^−/−^ mice after *S. japonicum* infection (Fig. 5E). Additionally, RNA-seq was performed on livers from CCl_4_-challenged *Cep72*^−/−^ mice. Analysis of the RNA-seq data from this CCl_4_-induced liver fibrosis model identified 147 upregulated and 81 downregulated genes (Supplementary Fig. S2A). The upregulated genes were primarily enriched in pathways associated with wound healing and the regulation of natural killer cell chemotaxis, while the downregulated genes were mainly enriched in pathways such as negative regulation of gluconeogenesis (Supplementary Fig. S2B). Heatmap analysis revealed that fibrosis-related genes were markedly increased in *Cep72*^−/−^ mice following CCl_4_ injection compared with the control group (Supplementary Fig. S2C). These results revealed that CEP72 deletion activates the expression of inflammatory and fibrosis genes in liver fibrosis.

### CEP72 deficiency promotes TNF-α transcription through regulating EGR1 expression

**Fig. 6:**
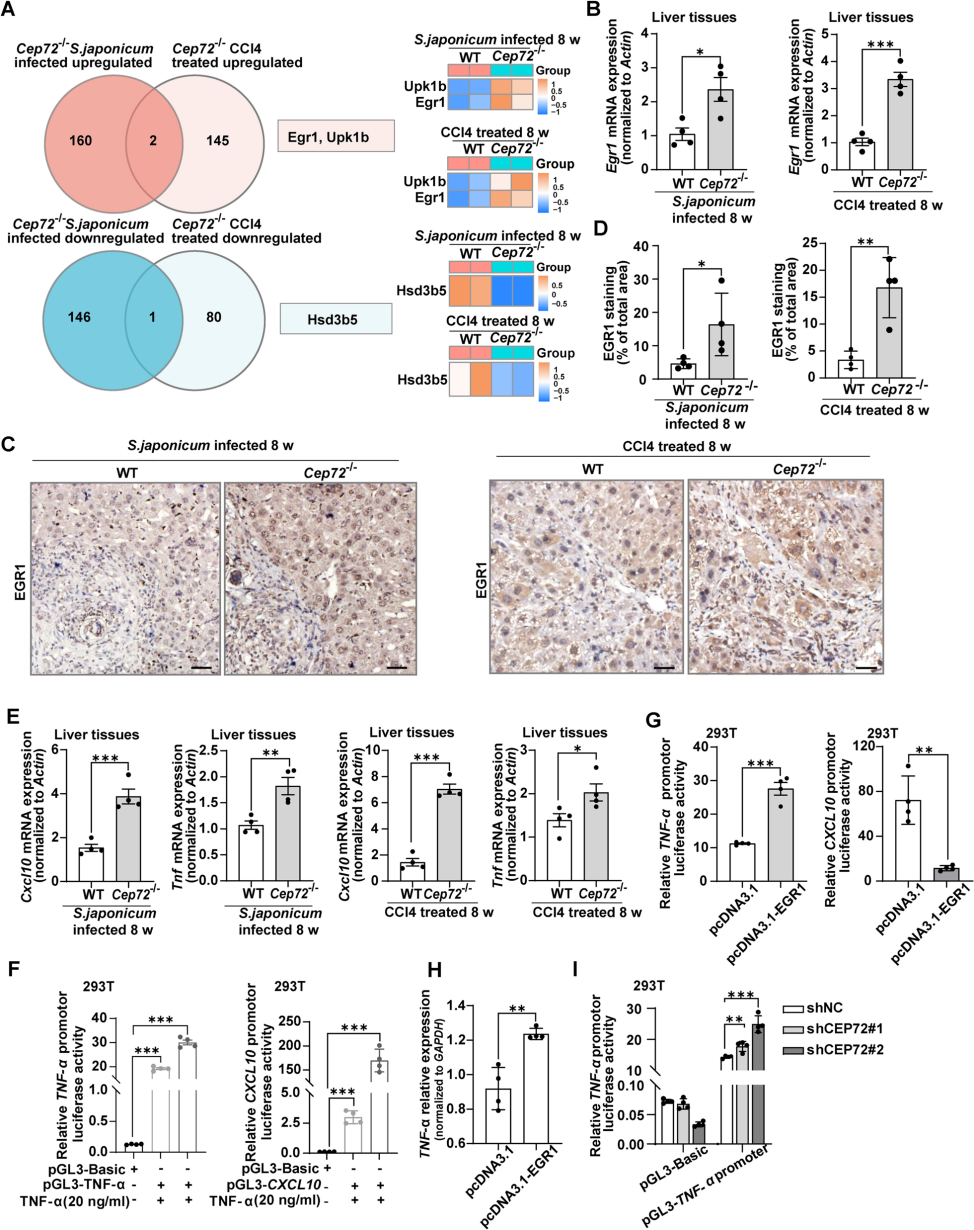
CEP72 deficiency promotes TNF-α transcription through upregulation of EGR1. **A** Venn diagrams showing the overlap of upregulated and downregulated differentially expressed genes (DEGs) between WT and *Cep72*^−/−^ mice after *S. japonicum* infection or CCl_4_ treatment (left), and heatmaps depicting the expression of shared upregulated DEGs (*Egr1* and *Upk1b*) and the shared downregulated DEG (*Hsd3b5*) in each condition (right). **B** qRT-PCR analysis of *Egr1* mRNA expression in livers from WT and *Cep72*^−/−^ mice following *S.japonicum* infection or CCl_4_ treatment. **C** Representative immunohistochemical staining for EGR1 in liver sections from WT and *Cep72*^−/−^ at 8 weeks after *S. japonicum* infection or CCl_4_ treatment (scale bars, 50 μm). **D** Quantification of EGR1-positive areas from immunohistochemical staining, analyzed using Image J from multiple randomly selected fields. **E** qRT-PCR analysis of *Tnf-α* and *Cxcl10* mRNA expression in WT and *Cep72*^−/−^ mice after *S.japonicum* infection or CCl_4_ treatment. **F** Dual-luciferase reporter assays of *TNF-α* and *CXCL10* promoters in 293T cells. **G** Dual-luciferase reporter assays of *TNF-α* and *CXCL10* promoters in 293T cells transfected with pcDNA3.1 or pcDNA3.1-EGR1. **H** qRT-PCR analysis of *TNF-α* mRNA expression in 293T cells overexpressing EGR1. **I** Dual-luciferase reporter assay of the *TNF-α* promoter in 293T cells transfected with control shRNA (shNC) or CEP72-targeting shRNAs (shCEP72#1 and shCEP72#2). Data are presented as the mean ± SD (*n* = 4 mice per group for in vivo experiments) and are representative of at least three independent experiments. Statistical analyses were performed using an unpaired Student’s *t*-test or one-way ANOVA. *P < 0.05; **P < 0.01; ***P < 0.001; ****P < 0.0001; ns, not significant

To further elucidate the underlying mechanisms by which CEP72 deletion aggravates liver fibrosis in *S. japonicum*- and CCl_4_-induced models, we performed an integrated analysis of RNA-seq data from the two models. Overlap analysis revealed a shared increase in the expression of early growth response-1 (*Egr1*) and uroplakin 1b (*Upk1b*) in *Cep72*^−/−^ mice with *S. japonicum*- or CCl_4_-induced liver fibrosis (Fig. 6A). Additionally, one gene was consistently downregulated in both models in *Cep72*^−/−^ mice (Fig. 6A). We focused on *Egr1* because it is a key transcription factor in fibrotic diseases; upon induction by environmental stimuli, EGR1 regulates the expression of genes involved in fibrogenesis [18]. We therefore examined *Egr1* expression in both *S. japonicum*- and CCl_4_-induced liver fibrosis in *Cep72*^−/−^ mice. qRT-PCR analysis showed that *Egr1* mRNA expression was significantly upregulated in *Cep72*^−/−^ mice following *S. japonicum* infection or CCl_4_ treatment (Fig. 6B). Furthermore, IHC staining showed increased EGR1 protein levels in *Cep72*^−/−^ mice after *S. japonicum* infection or CCl_4_ treatment, in line with our transcriptomic and qRT-PCR findings (Fig. 6C, D).

Previous studies have demonstrated that EGR1 binds to the promoters of key fibrogenic genes, such as *Tgf-β3*, *Pdgf-α*, *Pdgf-b*, *Col1α*, *Pai-1*, and *Timp3*, thereby increasing the expression of these downstream genes [18]. However, the role of EGR1 in regulating the expression of inflammation-related genes remains poorly understood. To investigate whether EGR1 activates the transcription of pro-inflammatory genes, we focused on *Tnf-α* and *Cxcl10* for subsequent analysis. This selection was guided by our heatmap analysis, which identified these two genes as the most markedly altered inflammatory genes in both WT and *Cep72*^−/−^ mice following *S. japonicum* infection or CCl_4_ treatment. Additionally, qRT-PCR results also indicated that CEP72 deficiency significantly upregulated the mRNA expression of *Tnf-α* and *Cxcl10* in the livers of *S. japonicum*-infected or CCl_4_-treated mice (Fig. 6E). Subsequently, we generated luciferase reporter plasmids by cloning the *TNF-α* and *CXCL10* promoters into the pGL3-Basic vector (Fig. 6F). EGR1 overexpression significantly induced luciferase activity driven by the expression of the *TNF-α* promoter–luciferase construct but inhibited the activity of the *CXCL10* promoter–luciferase construct (Fig. 6G; Supplementary Fig. S3A, B). These data indicate that EGR1 mediates the upregulation of *TNF-α* transcription. Moreover, qRT-PCR analysis demonstrated that overexpression of EGR1 significantly increased the expression of *TNF-α* at the mRNA level (Fig. 6H).

To further clarify the role of CEP72 in promoting *TNF-α transcription*, two short hairpin RNAs for *CEP72* (shRNA1 and shRNA2) were designed to target distinct regions of *CEP72*, and both efficiently reduced CEP72 expression. Subsequently, *CEP72* knockdown significantly enhanced *TNF-α* promoter-driven luciferase activity in 293T cells, as determined by the luciferase reporter assay (Fig. 6I; Supplementary Fig. S3C). Taken together, these results suggest that CEP72 may promote TNF-α transcription, at least in part, by regulating EGR1 expression.

## Discussion

This study identifies CEP72 as an important regulator of liver fibrosis. We found that CEP72, a centrosomal protein essential for maintaining centrosome structural integrity, is markedly upregulated in fibrotic livers from both schistosomiasis and CCl_4_-induced hepatopathy. Interestingly, *Cep72*^−/−^ mice display more severe liver fibrosis after infection with *S. japonicum* or induction with CCl_4_, in comparison with WT mice. These findings indicate that CEP72 is not simply a passive marker of fibrogenesis but may exert a protective role in restraining the progression of liver fibrosis.

Previous studies have primarily implicated CEP72 in tumor biology. High expression of CEP72 drives the progression and pathogenesis of various human cancers, such as gastric cancer (GC) and urothelial carcinoma of the bladder (UCB) [19]. The specific mechanism behind this is that overexpression of CEP72 leads to abnormal division of cancer cells, promoting their invasiveness and chromosomal instability. Furthermore, CEP72 is involved in the centrosomal protein CG-NAP and γ-TuRC localization, as well as in the microtubule organizing activity of the centrosome [20]. Given that centrosomes are crucial for cell-cycle transitions (G1–S, G2–M, and metaphase–anaphase) [21], disturbances in centrosome number or function can perturb mitotic spindle assembly and disrupt cell-cycle control. Precise regulation of the cell cycle is essential for tissue homeostasis and regeneration, whereas sustained cell-cycle dysregulation has been linked to fibrotic remodeling. In renal fibrosis, mild injuries could stimulate the proliferation of renal cells so as to compensate the renal cell loss and restore renal function. Nonetheless more severe or repeated injuries lead to cell-cycle dysregulation of renal cells, promoting renal fibrosis [22]. Bangen’s research [23] further confirmed that Cyclin E1 (CcnE1), a regulatory subunit of the Cyclin-dependent Kinase 2 (Cdk2) and a key driver of cell cycle reentry, regulates liver inflammation and fibrosis. Inhibiting mRNA expression of CcnE1 by siRNA in hepatocytes and nonparenchymal cells can ameliorate liver fibrosis and inflammation. In this context, our observation that Cep72 is highly expressed in fibrotic livers yet its deletion exacerbates fibrosis suggests that CEP72 may be required to maintain centrosome integrity and proper cell-cycle dynamics in liver cells. Loss of CEP72 could favor maladaptive cell-cycle regulation during chronic injury, thereby predisposing the liver to exaggerated fibrotic responses.

Hepatic fibrosis is a prototypical consequence of chronic inflammation. Persistent liver injury and hepatocyte cell death trigger inflammatory responses, which subsequently drive fibrogenesis by activating HSCs that transdifferentiate toward matrix-producing myofibroblasts [8]. During homeostasis, the liver tissue is scattered with immune cells that are distributed throughout the parenchyma but enriched in periportal regions [24]. Following liver injury, chemokines coordinate the recruitment and positioning of immune cells. In liver fibrosis caused by schistosomiasis, solution egg antigens (SEAs) released from deposited eggs induce T lymphocytes to produce key inflammation cytokines, such as TGF-β, TNF, interleukin (IL)-6, IL-10, and IL-13, ultimately leading to HSC activation, periuvular granuloma formation, and chronic liver fibrosis [3]. In our study, GO analysis of upregulated genes in schistosomiasis-infected or CCl_4_-injected *Cep72*^−/−^ mice revealed that genes associated with cell activation and inflammation are significantly enriched. Especially pro-inflammation cytokines such as *Tnf*, *Cxcl10*, *Il6*, and *Il1a*, were significantly upregulated in *Cep72*^−/−^ livers compared with WT controls, supporting the notion that CEP72 deficiency amplifies inflammatory signaling linked to HSC activation and fibrogenesis.

EGR1 is a Smad-independent intracelluar mediator of TGF-β signaling and has been implicated in multiple fibrotic diseases. Aberrant EGR1 expression and function have been associated with experimental models of fibrosis and human disorders such as idiopathic pulmonary fibrosis and scleroderma [25, 26]. Herein, we found that EGR1 expression was increased in both *S. japonicum*- and CCl_4_-treated *Cep72*^−/−^ mice compared with the control. Furthermore, we identified *TNF-α* and *CXCL10* as the most strongly altered inflammatory genes in our RNA-seq and heatmap analyses. Functional assays demonstrated that EGR1 directly promotes TNF-α transcription: EGR1 overexpression increased TNF-α promoter-driven luciferase activity and elevated TNF-α mRNA levels, although its effect on the CXCL10 promoter appeared more complex. Given that TNF-α is a critical mediator of liver injury, fibrosis, and nonalcoholic steatohepatitis [27, 28], and that genetic deficiency of TNF-α or its receptors (TNFR1/TNFR2) ameliorates bile duct ligation-induced fibrosis and steatohepatitis-associated steatosis and fibrosis [27], our data support a model in which CEP72 deficiency potentiates fibrogenesis at least in part by enhancing EGR1–TNF-α signaling.

In summary, we show that CEP72 deficiency exacerbates liver fibrosis in both *S. japonicum*- and CCl_4_-induced models and identify a CEP72–EGR1–TNF-α axis linking centrosome-associated regulation to inflammatory signaling. CEP72 appears to act as a protective factor by limiting EGR1-dependent TNF-α transcription and restraining excessive inflammation, revealing a noncanonical antifibrotic role for this centrosomal protein. Further work, particularly using cell-type-specific models, is needed to clarify how CEP72 controls EGR1–TNF-α signaling in distinct hepatic cell populations and to assess whether this pathway can be exploited therapeutically.

## Supplementary Information

Additional file 1. Figure S1. CEP72 expression is upregulated in CCl_4_-induced liver fibrosis. (A) Expression of *Cep72* in CCl_4_-induced liver fibrosis from GSE222576. (B) *Cep72* transcript levels were assessed using an in-house RNA-seq data obtained from fibrotic mouse liver tissues following 8-week CCl_4_ injection. (C) *Cep72* mRNA expression level was assessed in liver tissues from control and mice injected with CCl_4_ for 8 weeks by qRT-PCR. (D) ACTA2 staining and CEP72 staining (all scale bars, 50 μm) of liver sections from the indicated groups (control, 6 w, and 8 w CCl_4_ injected). (E) H&E staining of liver sections from WT and *Cep72*^−/−^ mice. (F) CEP72 staining of liver sections from *S. japonicum*-infected WT and *Cep72*^−/−^ mice (scale bars, 50 μm). Data are presented as mean ± SD. Statistical significance was assessed by one-way ANOVA for multiple-group comparisons (A) and by unpaired two-tailed Student’s *t*-test for two-group comparisons (B, C). *P < 0.05; **P < 0.01; ***P < 0.001; ****P < 0.0001; ns, not significant.

Additional file 2. Figure S2. Pro-fibrotic and pro-inflammatory genes were up-regulated in Cep72^−/−^ mice in CCl_4_-induced liver fibrosis according to RNA-seq. (A) Volcano plots illustrate the up- or downregulated genes in WT and Cep72^−/−^ mice treated with CCl_4_, based on RNA-seq data. (B) Pathway analysis of RNA-seq data showing the differentially expressed upregulated genes and downregulated genes in WT and Cep72^−/−^ CCl_4_ injected mice. (C) Heatmaps of fibrosis-related genes and inflammatory factors are shown according to RNA-seq of WT and Cep72^−/−^ CCl_4_ injected mice.

Additional file 3. Validation of EGR1 overexpression and CEP72 knockdown in 293T cells. (A) qRT-PCR analysis of *EGR1* mRNA expression in 293T cells transfected with empty vector (pcDNA3.1) or EGR1 expression plasmid (pcDNA3.1-EGR1). (B) Western blot analysis of EGR1 protein levels in 293T cells transfected with pcDNA3.1 or pcDNA3.1-EGR1; GAPDH was used as a loading control. (C) qRT-PCR analysis of *CEP72* mRNA expression in 293T cells transfected with control shRNA (shNC) or CEP72-targeting shRNAs (shCEP72#1 and shCEP72#2). Data are presented as mean ± SD from three independent experiments. Statistical significance was assessed using an unpaired two-tailed Student’s *t*-test for two-group comparisons (A) and one-way ANOVA for multiple-group comparisons (C). *P* < 0.05 was considered statistically significant (**** *P* < 0.0001).

## Data Availability

The RNA-Seq FASTQ files were deposited in NCBI's Gene Expression Omnibus (GEO) (GSE318357), accessible password is **ylidekwmtvgltwt** **.**

## Abbreviations

CEP72: Centrosomal protein 72
ALT: Alanine aminotransferase
AST: Aspartate aminotransferase
HSCs: Hepatic stellate cells
ECM: Extracellular matrix
WT: Wild-type
Col1a1: Collagen I
Acta2: Actin alpha 2
Tgf-β1: Transforming growth factor beta 1
Egr1: Early growth response protein 1
