# Supplementary figures and images for: Centrosomal protein 72 deficiency exacerbates liver fibrosis induced by Schistosoma japonicum infection
Source: Parasit Vectors. 2026 May 5;19:258. doi: 10.1186/s13071-026-07372-7 (PMC13289381; doi:10.1186/s13071-026-07372-7)

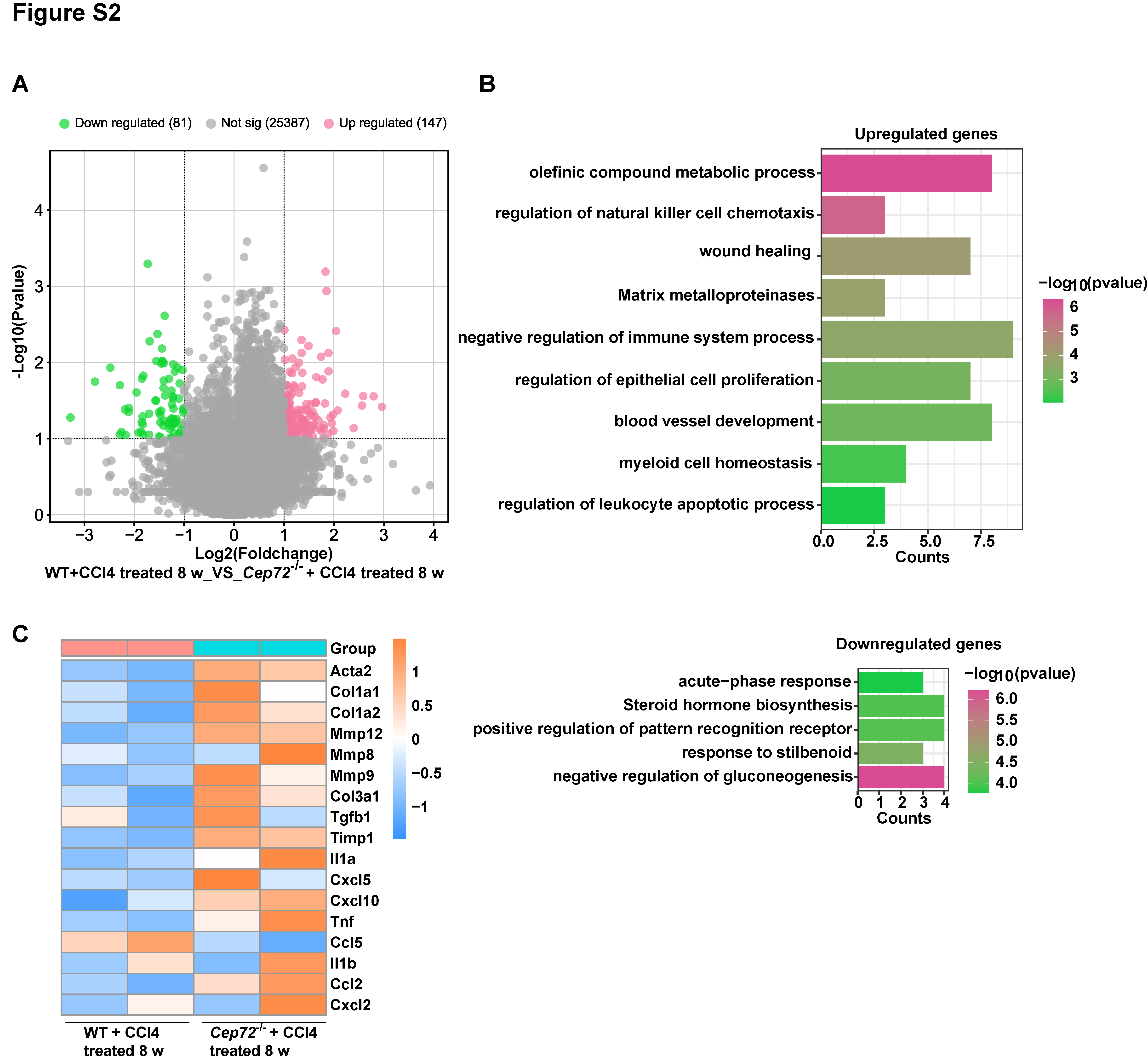

Supplement: Supplementary file 2 — Additional file 2. Figure S2. Pro-fibrotic and pro-inflammatory genes were up-regulated in Cep72−/− mice in CCl4-induced liver fibrosis according to RNA-seq. (A) Volcano plots illustrate the up- or downregulated genes in WT and Cep72−/− mice treated with CCl4, based on RNA-seq data. (B) Pathway analysis of RNA-seq data showing the differentially expressed upregulated genes and downregulated genes in WT and Cep72−/− CCl4 injected mice. (C) Heatmaps of fibrosis-related genes and inflammatory factors are shown according to RNA-seq of WT and Cep72−/− CCl4 injected mice. [file 13071_2026_7372_MOESM2_ESM.tif]

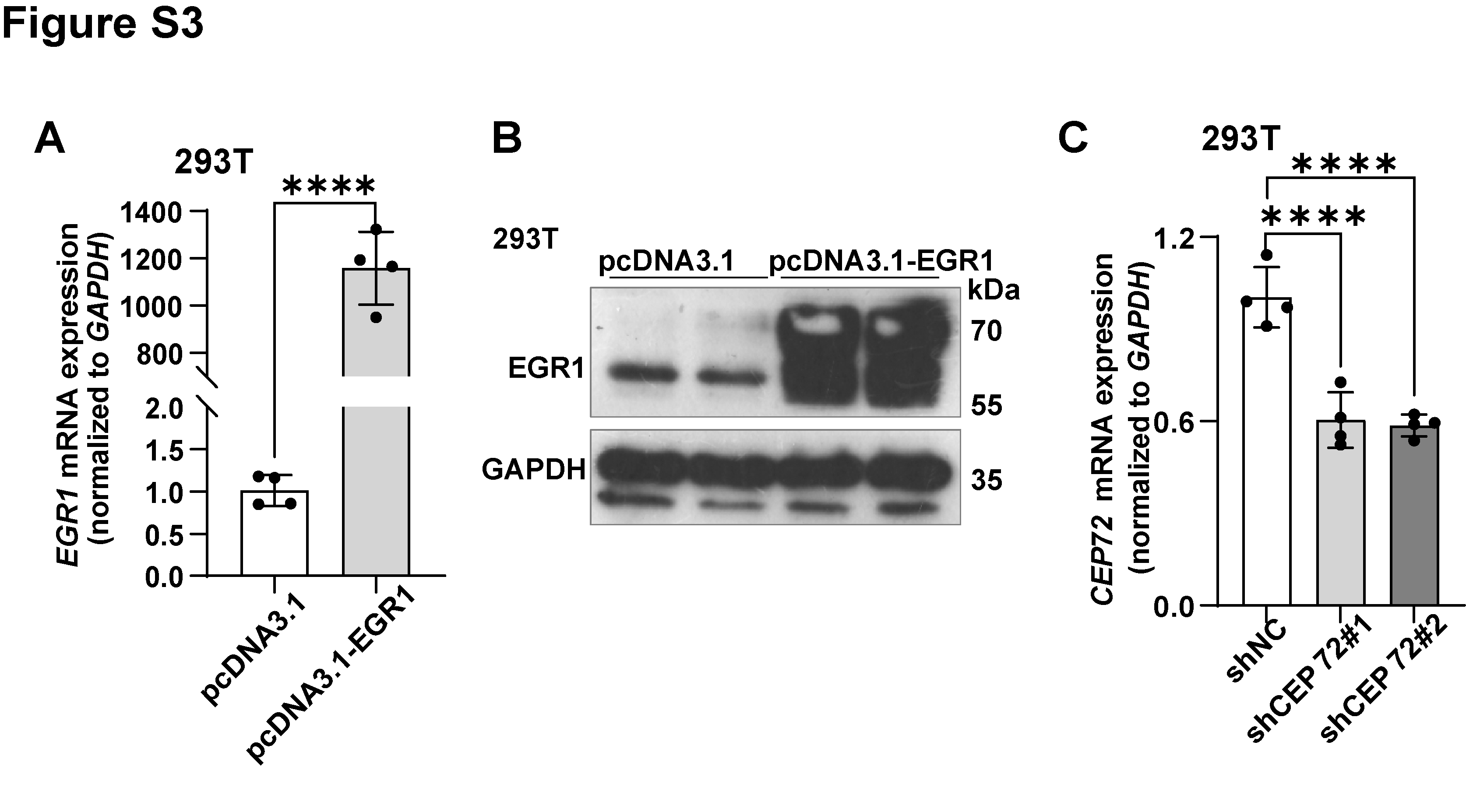

Supplement: Supplementary file 3 — Additional file 3. Validation of EGR1 overexpression and CEP72 knockdown in 293T cells. (A) qRT-PCR analysis of EGR1 mRNA expression in 293T cells transfected with empty vector (pcDNA3.1) or EGR1 expression plasmid (pcDNA3.1-EGR1). (B) Western blot analysis of EGR1 protein levels in 293T cells transfected with pcDNA3.1 or pcDNA3.1-EGR1; GAPDH was used as a loading control. (C) qRT-PCR analysis of CEP72 mRNA expression in 293T cells transfected with control shRNA (shNC) or CEP72-targeting shRNAs (shCEP72#1 and shCEP72#2). Data are presented as mean ± SD from three independent experiments. Statistical significance was assessed using an unpaired two-tailed Student’s t-test for two-group comparisons (A) and one-way ANOVA for multiple-group comparisons (C). P < 0.05 was considered statistically significant (**** P < 0.0001). [file 13071_2026_7372_MOESM3_ESM.tif]
